# Effects of lower-extremity explosive strength on youth judo athletes adopting different types of power-based resistance training

**DOI:** 10.3389/fphys.2023.1065036

**Published:** 2023-03-15

**Authors:** Ruiyin Huang, Mingyang Zhang, Linjie Huang, Zilong Chen, Yong Mo, Yuhua Gao

**Affiliations:** ^1^ Graduate School, Guangzhou Sport University, Guangzhou, China; ^2^ School of Athletic Training, Guangzhou Sport University, Guangzhou, China

**Keywords:** strength training, resistance type, pneumatic resistance, vertical jump, linear speed

## Abstract

**Objective:** The present study compared the effects of two different resistance types (pneumatic resistance and free weight) of 6-week squat training on the performance for young female judo athletes in linear speed and vertical jump by utilizing the maximum power of each set of squats in each training session as the monitoring vehicle. Monitoring data were used to assess the effects and trends of the two resistance types on 70% 1RM weight-bearing during the 6-week intervention training.

**Methods:** In a 6 weeks squat training (2 reps/week with a constant load), 23 adolescent female judo athletes (Age span: 13–16 years, 14.58 ± 0.96) were randomly selected and then divided into the traditional barbell (FW) group (*n* = 12) and the pneumatic resistance (PN) (*n* = 11) group according to different resistance types (free weight and pneumatic resistance), with 10 in FW group and 9 in PN group actually completed the study. Before and after training, the 30-m Sprint time (T-30M), vertical jump height and relative power (countermovement jump, static-squat jump, and drop jump), reactive strength index (DJ-RSI), and maximal strength were assessed. One-Way ANOVA was used to examine the pre-test differences of groups (FW and PN). A 2-factor mixed-model analysis of variance was used to examine the independent effects of group (FW and PN) and time (pre and post) on each dependent measure. Scheffe *post hoc* comparisons were used to examine the differences. Pre- and post-experimental differences between the two groups were analyzed using independent samples *t*-tests and magnitude-based inferences (MBI) derived from their *p* values, and effect statistics were applied to compare the pre- and post-changes exhibited by each group to identify the potential beneficiary groups.

**Results:** The PN group outperformed the FW group in terms of maximal power output per training session (822.5 ± 55.22 vs. 927.42 ± 48.15, conventional vs. pneumatic, *p* < 0.001, effect size = −2.02). After 6 weeks of training, the FW group showed significant increases in vertical jump height and relative strength (CMJ, SJ, DJ), with no significant gains observed in T-30 and maximal strength. The PN group showed significant improvements in maximal strength; however, no significant improvements were observed in the other tests. In addition, there was no significant difference in DJ-RSI between the two groups before and after training.

**Discussion:** At 70% weight bearing, free weight resistance appears to be more conducive to vertical jump growth, while pneumatic resistance appears to be more conducive to maximal strength gains; however, the maximal strength gains from pneumatic resistance may not be well applied to athletic performance. In addition, the body adapts more quickly to pneumatic resistance than to free weight resistance.

## Introduction

Resistance is a force generated during the relative motion of an object in the opposite direction of motion. Gravity, inertia, friction, fluid resistance, and elasticity are the most typical causes of resistance in strength training (Harman, 1994), Resistance training is classified into 3 categories: constant resistance, regulated resistance, and variable resistance (McMaster et al., 2009). Constant resistance is defined as a constant external load throughout the range of motion; regulated resistance is defined as a constant velocity load that provides a controlled speed throughout the range of motion; whereas fluid-based resistance is similar to regulated and variable resistance and has two types: hydraulic resistance and pneumatic resistance (McMaster et al., 2009). Variable resistance is defined as the type of resistance that changes during exercise (Frost et al., 2010).

As athletes’ training needs change at different stages, coaches usually organize athletic training in a linear, non-linear, or wave-like manner, i.e., using incremental, irregular, or alternating high and low loads to apply repetitions, weights, and intervals to athletes (Monteiro et al., 2009; Prestes et al., 2009; Painter et al., 2012; Bartolomei et al., 2014); However, coaches often overlook the replacement of resistance training types. Comparative studies of different types of resistance training have focused on variable resistance *versus* free weight resistance. Free weight resistance is an effective method for improving an athlete’s strength (Ebben and Jensen, 2002; Bellar et al., 2011; Andersen et al., 2015), however, due to the inertial limitations of weighted objects, it is inevitably limited by inertia and reduces the muscle effort required to complete repetitions in the range of motion (Frost et al., 2008). Moreover, if the weighted object is not thrown during the second half of the range of motion, the athlete must actively decelerate the weighted object (Newton et al., 1996). A few current studies of aerodynamic resistance *versus* free weight are mostly based on the kinematics and dynamics of different weight-bearing intervals (Newton et al., 1996; Peltonen et al., 2013; Frost et al., 2016). Frost et al. (2008) demonstrated that aerodynamic resistance allows for a good level of force output during training, which may lead to a greater degree of performance transfer during high-speed movements, although emphasizing that this argument is highly inferred based on the evidence. However, McMaster et al. (2009) argued that free weight resistance is a better stimulus because it simulates real-life movements and provides natural muscle tissue coordination (McMaster et al., 2009). Muscle contraction during exercise is governed by Newton’s second law of motion and the law of conservation of momentum, and the effect of this control on the associated kinetics, kinematics, and muscle activity is dependent on the type of resistance applied (Frost et al., 2008).

Previous research has shown that the vertical jump may be used as an index to evaluate the explosive output of lower limbs and the dynamic performance of lower limb muscles (Markovic et al., 2004). Additionally, the heights achieved on the countermovement jump (CMJ) and drop jump (DJ) tests were considered to be indicative of the effectiveness of the slow and fast stretch-shortening cycles (SSCs), respectively; however, the Squat Jump (SJ) performance was primarily considered to be representative of the initial strength capacity. Through a correlation analysis of 21 adult rugby players, Furlong, Laura-Anne M et al. (2021) discovered a correlation between the 30 M sprint time and CMJ, SJ, and DJ. Furthermore, explosive force is the ability to work quickly in a short period, and power as a physical quantity to evaluate how quickly work done over time, may be interpreted as the human explosive force, which is the body’s ability to work externally.

In addition, training monitoring in this study was used to: 1) observe the change in explosive power gain by different resistance types by monitoring the change in maximum output power values for each training session in both groups; 2) indirectly reflect the time required for athletes to adapt to their corresponding resistance type by combining the participative fatigue scale (RPE) and the important inflection points of power for both resistance types. Research has demonstrated the feasibility of using kinetic data as real-time feedback information to athletes and for monitoring purposes, e.g., velocity-based resistance training uses movement speed values fed by linear sensors to keep athletes moving in specific speed intervals as much as possible to achieve training objectives (Jovanović and Flanagan, 2014). Power was assumed to be better indicator of the difference in explosive power between the two groups of athletes than speed in previous studies comparing free weight resistance with pneumatic resistance: whether monitored for a single training movement or the entire intervention (Frost et al., 2010; Franchini et al., 2011; Frost et al., 2016). In this study, power was chosen as the indicator for real-time feedback information and monitoring.

Judo is an open, physically demanding sport that also requires a high degree of technical and tactical skills to compete and win. Judo competition is characterized by its short duration, high intensity, and intermittent nature (Franchini et al., 2011); the key to victory is the application of explosive special movements to subdue opponents, and this short burst of energy is mainly provided by the anaerobic metabolism. Findings of Sikorski et al. (1987) corroborate the aforementioned statement. In addition, judo is about overcoming rigidity with softness and flexibility, so the external force that the athlete resists during the competition is constantly changing; it may be beneficial for the athlete to try different types of resistance during the training process.

In summary, the purpose of this study was to examine the effects of 6 weeks of deep squat training with two different resistance types on the linear velocity and vertical jump performance of female youth judo athletes by using the maximum power of each squat set in each training session as a monitoring vehicle and to determine whether improvements in strength or peak power were transferable to the athletic performance by observing changes in various linear velocity and vertical jump parameters. In addition, the study indirectly assessed the difference in adaptation to 70% 1 RM load between the two resistance types during the 6-week intervention training

## Methods

### Study design

In this single-blind, randomized controlled intervention trial, participants were blinded to the purpose and intent of the study. The study was conducted during the off-season (November–December 2021) after the competition period of September 25–28, following which the women entered a 1-month recovery period. During the recovery period, all participantsparticipant were trained in the deep squat technique and pneumatic resistance adaptation. All participants who met the inclusion criteria were recruited and randomly assigned into two groups: the free weight group and the pneumatic resistance group. The randomization program was performed by a person who was blinded to the study by using the random number table method; the participantsparticipant assigned to the pneumatic resistance group constituted the intervention group, whereas those assigned to the free weight group constituted the control group. All participantsparticipant completed 5 baseline tests prior to randomization, of which the jump test (CMJ, SJ, and DJ) and the 30 M-sprint test served as the primary indicators to assess explosive lower-extremity strength. The maximum strength test index was used as a secondary indicator in this study. In addition, the training movement for this intervention was performed with weighted squats. Moreover, to avoid unnecessary interference from differences in the squatting movement patterns, the Functional Movement Screen (overhead squat) was used as the screening index for participants (FMS scores < 1 not included in the participants). A total of 4 test days (2 pre-test days and 2 post-test days) were scheduled, taking into account the risk of interference with energy supply and the fatigue effect of multiple test items. All tests (pre- and post-tests) were performed in the same order, at the same intervals, at the same locations, and with the same 20-min standardized warm-up performed on each test day. A standardized 20-min warm-up (content: dynamic stretching, muscle activation) before each training session and a standardized 10-min cool-down (content: static stretching) after the session.

### Participants

**FIGURE 1:**
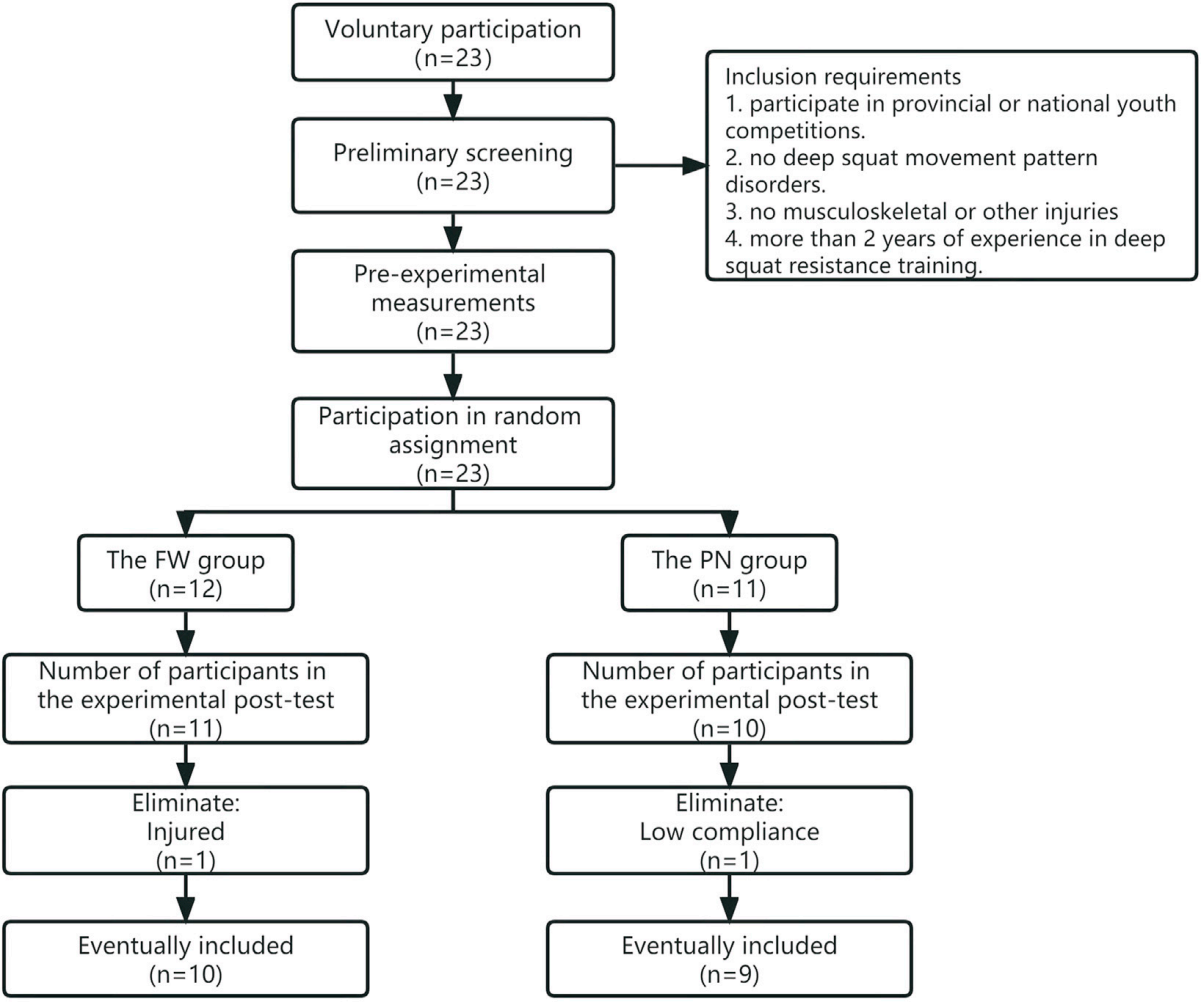
Population screening flowchart.

The study was conducted in strict accordance with the requirements of the Regional Medical and Health Research Ethics Committee, as per the Declaration of Helsinki and following the international ethical standards. All participants (or participants’ guardians) signed written informed consent after receiving all relevant information about the study. The Ethics Committee of Guangzhou Sports Institute approved the study protocol. As shown in Figure 1, a total of 23 adolescent female judo athletes from the sports schools in the Guangdong Province volunteered to participate in this trial and randomly assigned into two groups: the free weight group (*n* = 12) and the pneumatic resistance group (*n* = 11). All participants passed the FMS screening test (score > 1), albeit they were unable to complete the post-test due to low compliance (*n* = 1), illness or injury (*n* = 1), or missed test visits (*n* = 2). A total of 19 participants (*n* = 19, age: 14.58 ± 0.96 years) completed all phases of the intervention training and testing and were included in the study (FW: *n* = 10; PN: *n* = 9). The inclusion criteria were as follows: participation in provincial or national youth competitions, no deep squat movement pattern disorders, no musculoskeletal or other injuries, and at least 2 years of experience in deep squat resistance training. Each group was supervised and protected by at least 1 athletic trainer during each intervention training session. Considering that female subjects in this age group are undergoing a developmental period of rapid changes in body size, shape and composition, and that there may be significant effects of their biological maturity on training response, differences in physiological maturity in the subgroup population were determined by testing subjects’ age of peak height velocity (APHV) (Balyi and Hamilton, 2004).

### Training program

All participantparticipants in the experimental intervention completed a 6-week strength training intervention (2 interventions per week on Saturday and Thursday; the participantsparticipant underwent a daily judo-specific technical training schedule during the week in addition to the experimental intervention). Each strength training intervention was scheduled in the afternoon (2:00–3:30 p.m. on Thursday and 4:00–5:30 p.m. on Saturday), with 48-h interval between the training interventions, and none were affected by holidays. The interventions for both the pneumatic resistance and free weight groups were conducted at the same time in the gym of the Guangdong Provincial Sports School, with at least 2 athletic trainers per group for protection, supervision, and data collection during the training sessions. All participants completed the maximum strength (1 RM) test in the free weight (barbell) form prior to the intervention, and after determining the 1 RM weight, both the free weight and pneumatic resistance groups were trained at 70% of the 1 RM weight (the load weight of the pneumatic resistance group was adjusted synchronously with the 1 RM weight measured in the barbell form). After a standardized warm-up process for each training session, complete 4 sets (interval between sets: 90 s–120 s) of 8 reps of behind-the-neck squats. Both groups were trained with a constant load (i.e., similar training movement, load weight, number of repetitions, sets, and intervals) during the 6-week intervention course.

During the training session, one athletic trainer recorded the maximum power output value in each set of behind-the-neck squats for the corresponding participants in real time as a monitoring vehicle for this experiment. The free weight group used a linear transducer (GymAware Power Tool; Kinetic Performance Technologies, Australia) fixed 60-cm to the right of the center of the barbell to collect the maximum power output value for each squat *via* Bluetooth connection to an iPad. The pneumatic resistance group recorded the maximum power output value on the left-front display of the pneumatic squat device (KEISER, POWER RACK, MODEL 3110, Germany) on the left-front display. Another athletic trainer was responsible for protecting and checking the range of the participant’s squatting movements, and any repetitions that did not meet the movement requirements were not recorded. The power output value for each repetition was fed back to the participant (visually or audibly) in real time, and the participants were strongly informed verbally during the process to perform maximal voluntary contractions with the maximal effort intent to complete the centrifugal phase slowly. In addition, the participants were asked to complete a subjective fatigue evaluation scale (RPE) for fatigue monitoring at the end of each training session.

### Testing program

The pre-test and post-test were administered 1 week before and 7 weeks after the intervention training, respectively. For the pre-test, the participants were allowed a week’s time period before the test to allow them to familiarize themselves with the test items. During the testing week (Thursday and Saturday), the participants entered the gym and the perimeter track for two unit tests, namely, the jumping ability test and the sprinting ability test. The order of the test items, the sequencing of participants, and the scheduling of items for the testers were the same between the pre-test and post-test. All participants were asked to maintain a regular diet and adequate sleep, as well as to avoid strenuous exercise prior to undertaking the tests.

### Outcome measures

#### Body composition

The participants were required to complete the body composition test before the warm-up and asked to sleep adequately and eat normally in the day before the test, and no physiological cases were noted during the body composition test. After determining the participants’ fasting and dehydration status for 4 h, the body composition test was performed using a bioelectrical impedance analyzer (InBody 370, Biospace, Soul, Korea) for determining the weight, and body fat percentage. Height was measured using a measuring tape, and the participants were asked to stand barefoot and upright against a wall for measurement; Participants’ sitting height and leg length were measured using the anterior superior iliac spine as the base point. Data of the Height, age, and sex were then entered into a bioelectrical impedance analyzer for uniform output. The body composition test data only served as the baseline characteristics for the randomized subgroups and were not used as data for before and after controls.

#### Vertical jump tests

The vertical jump test comprised 3 items, namely, countermovement jump (CMJ), drop jump (DJ), and static-squat jump (SJ). Three attempts were performed for each jump test and the relative peak power in the collected jump height and jump tests were collected as outcome metrics (the response strength index (RSI) was added to the DJ test as one of the outcome metrics). The same warm-up procedures, including fascial relaxation, dynamic stretching, and neuromuscular activation, were used for both the pre-and the post-intervention tests. All 3 jump tests were conducted in the gym (using the Australian Smart jump wireless test mat Fusion Sports Smart Jump Mat) in the following order: CMJ, DJ, and SJ. Three attempts were made for each test, with at least a 1-min interval between each attempt, and the best score among the 3 attempts was considered for data analyses. In addition, the interclass coefficient of the pre-test showed good confidence and reproducibility (heights of 0.966, 0.980, and 0.915 for CMJ, DJ and SJ, respectively; relative powers of 0.951, 0.987, and 0.978; and DJ-RSI of 0.940).

First, the reverse vertical jump (CMJ) was used to indirectly measure the athlete’s explosive lower body strength (Young, 1995; Markovic et al., 2004). The participants were asked to stand at the center of the jumping mat, place their hands on their hips, descend to the optimal jumping angle, jump upward to their maximum capacity, and land on the jumping mat in the initial position as far as possible after reaching the maximum height.

Second, the single DJ-RSI test was used to determine an athlete’s ability to show rapidly transition from the centrifugal to centripetal muscle contraction by testing their reactive jumping ability and to determine the athlete’s explosive ability to perform dynamic vertical jumps (Young, 1995; Flanagan et al., 2008). The participants were asked to stand with their hands on their hips, fall freely on a platform 20 cm above the ground, jump upward with the maximum effort taking as little time as possible in contact with the ground, and then fall freely to the jumping mat after reaching the maximum height.

Finally, the deep squat jump (SJ) test is commonly used to measure the explosive lower body strength (i.e., speed strength capacity) in athletes (Young, 1995; Markovic et al., 2004). The experiment was conducted in the form of a static SJ test, wherein the participants were asked to “standstill” in a semi-squatting position for 3 s before starting to jump upward. The arm position and body drop position during the test were kept consistent with that in the abovementioned jump test.

#### Sprint test

The 30 M sprint test was used to measure linear acceleration and linear velocity of the athletes (Stern et al., 2020). The participants were tested for 30 M of elapsed time by using the smart speed fusion sport (2 smart scans and 2 smart speeds pro, Australia). They were asked to start in a standing start position before the start line (0.5 M from the first light gate) and then decelerate and cushion after passing the second light gate at full power. Each participant made 3 attempts, with at least 3-min interval between each attempt. The 30 M sprint test was performed three times and the ICC showed good reliability and repeatability (ICC: 0.923).

#### Maximum strength test

The direct measurement method was used to test the maximum strength (Hoffman, 2011; Seo et al., 2012). After the standardized warm-up program, the participants were asked to warm-up with a 50% 1 RM load for 6–8 repetitions, during which they were verbally encouraged to complete the squat with the maximum effort possible. Considering the time and the number of participants allocated, the participants were monitored during the warm-up phase by using linear sensors, after which the load was adjusted according to the participants’ speed and effort made in squatting. A weight of approximately 75%–80% of 1 RM was first selected for 3–5 repetitions. The load was then increased to 90%–95% of the 1 RM weight for 1 repetition, and finally, a separate 1 RM attempt was made, with each weight increasing by 4 kg and decreasing by 2 kg. Then, 3–5 min of rest was allowed between all tests, and all participants were asked to achieve 1 RM in 3–7 attempts. Each group was monitored by more than 1 athletic trainer throughout the test. The participants were verbally encouraged and observed for squat depth (thighs parallel to the floor), and the last successful squat was recorded as the 1 RM weight. The subject adds weight incrementally in the direct measurement method, and in order ensure the subject’s maximum effort per squat, the maximum strength test is performed in only one attempt, so ICC calculations are not performed.

#### Statistical analyses

The measured variables included participants’ age, height, weight, body fat percentage, deep squat 1 RM; APHV, Competition class; 30 M sprint time, CMJ (height, peak power), SJ (height, peak power), and DJ (height, peak power, reaction strength index) at the baseline. The mean (standard deviation, SD) or median (range) was used as descriptive statistics to calculate the relative test-retest reliability by one-way random effects model with interclass coefficient (ICC) and 95% confidence interval (CI), and the reliability coefficient was generally considered lower than 0.4 to indicate poor reliability and greater than 0.75 to indicate good reliability (Wang et al., 2008). Standardized mean difference (SMD, mean change/baseline SD), Cohen’s D, and the smallest worthwhile change (SWC, 0.2 × baseline SD) were calculated for the 90% CI (Batterham and Hopkins, 2006; Boriani et al., 2007).

Shapiro-Wilk test was used for normality and Levene’s test was used for the chi-square test. One-Way ANOVA was used to examine the pre-test differences of groups (FW and PN). A 2-factor mixed-model analysis of variance was used to examine the independent effects of group (FW and PN) and time (pre and post) on each dependent measure. Scheffe *post hoc* comparisons were used to examine the differences. Two-sided *p*-values < 0.05 were considered statistically significant differences. Effect sizes (ES) were used to calculate pre and post-differences describing each dependent measure for both training groups. In addition, due to the small sample size, the intra-group ES strengths were interpreted using the general guidelines provided by Hedgesʼ g, i.e., strengths of 0.2, 0.5, and 0.8 correspond to small, medium, and large changes, respectively; the intra-group ES strengths were expressed using the partial Eta square, i.e., strengths of 0.01, 0.04, and 0.14 corresponded to small, medium, and large changes, respectively (Cohen, 2013) (Statistical power analysis for the behavioral sciences (2nd ed). Magnitude-based inference (MBI) was derived using *p*-values from independent sample t-tests and effect statistics to compare changes in each group before and after the experiment to identify potential beneficiary groups. Qualitative descriptions of each inference were derived based on the scale recommended by Hopkins (Hopkins, 2007): most likely, 0.5%; unlikely, 5%–25%; likely, 25%–75%; likely, 75%–95%; very likely, 95%–99.5%; and most likely 99.5%. The inferential statistics based on magnitude were calculated using a custom spreadsheet (Batterham and Cox, 2006). All other statistical procedures were analyzed using the statistical package jamovi 2.2.2.

## Results

As shown in Figure 1, twenty-three participants were screened and qualified to participate in this experimental intervention; however, four of these participants failed to satisfy the requirements of the experiment and were eliminated from the analysis. A total of 19 participants (FW:10 and PN:9) were statistically included in the metrics data. No descriptive variables or exercise parameters differed significantly between the FW and PN groups at baseline (Tables 1, 2).

**TABLE 1:** Participants’ demographic data.

Characteristics	FW	PN	*p*-value	SMD
Age (y)	14.3 ± 1.16	14.89 ± 0.60	0.19	0.60 (-0.16, 1.48)
Height (cm)	165.2 ± 6.61	164.7 ± 7.6	0.87	−0.07 (-1.05, 0.89)
Body mass (kg)	56.12 ± 7.6	64.6 ± 10.7	0.07	0.84 (-0.07, 1.97)
Fat mass (%)	15 ± 4.5	18.3 ± 6	0.19	0.60 (-0.31, 1.68)
APHV	1.29 ± 0.44	1.40 ± 0.25	0.49	0.31 (-0.47, 1.14)
Competition class (kg)	52.10 ± 7.29	56.11 ± 8.68	0.29	0.48 (-0.28, 1.34)

Note: Values are expressed as median (range) or mean (SD). The *p*-value in the *t*-test or Mann–Whitney *U* test denotes the between-group difference between the FW and PN groups; * represents a significant difference between the groups, and SMD stands for standardized mean difference.

**TABLE 2:** Between-group differences in each test index (pre-test).

Outcome	FW	PN	*p*-value	SMD	ICC
30-m	4.97 ± 0.195	5.10 ± 0.322	0.31	0.47 (-0.29, 1.33)	0.923
CMJ-High	29.60 ± 3.293	30.13 ± 5.397	0.54	0.12 (-0.67, 0.93)	0.966
CMJ-RP	40.92 ± 3.409	42.13 ± 4.797	0.73	0.28 (-0.49, 1.11)	0.951
SJ-High	28.59 ± 4.378	29.42 ± 5.749	0.77	0.16 (-0.63, 0.97)	0.980
SJ-RP	39.77 ± 4.355	41.55 ± 4.912	0.80	0.37 (-0.40, 1.21)	0.987
DJ-High	29.90 ± 4.470	30.66 ± 6.392	0.58	0.13 (-0.65, 0.95)	0.915
DJ-RP	41.44 ± 4.429	42.78 ± 5.755	0.42	0.25 (-0.53, 1.08)	0.978
DJ-RSI	0.74 ± 0.246	0.69 ± 0.208	0.61	−0.22 (-1.05, 0.56)	0.940
Back squat 1-RM (kg)	82.00 ± 10.995	83.78 ± 10.929	0.73	0.15 (-0.63, 0.97)	-

Note: Values are expressed as the median (range) or mean (SD). *p* values in the *t*-test or Mann–Whitney *U*-test indicate group differences between the FW and PN groups for the pre-test; SMD represents standardized mean differences; ICC:Intergroup correlation coefficients for pretests of each test item.

### Training data

**FIGURE 2:**
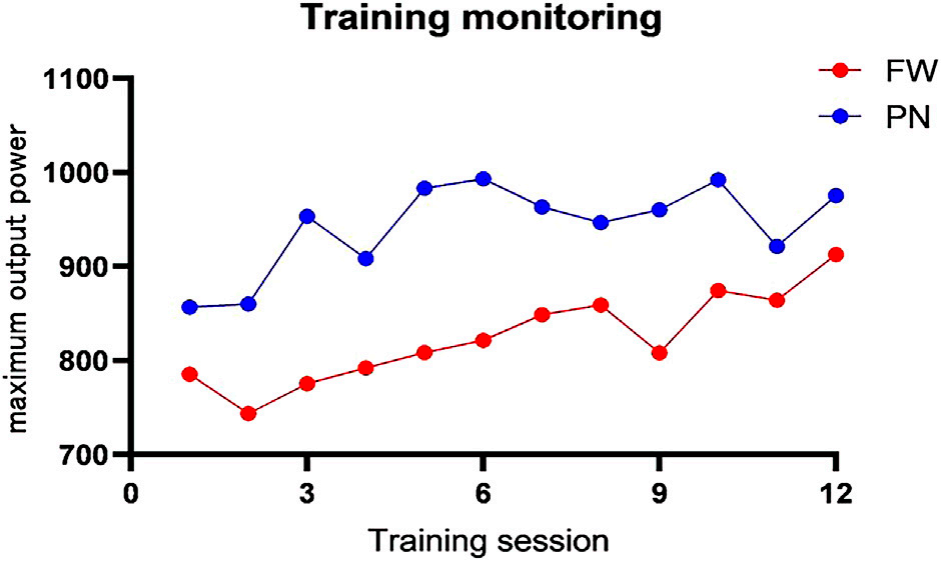
Training monitoring change curve (maximum output power).

As shown in Figure 2, the real-time monitoring data of the two groups were the maximum output power of each group in each training intervention, recorded in groups by training sessions. Participants in the PN group exhibited a greater mean maximum output per training session than those in the FW group in the 6-week linear incremental model (FW vs. PN; 822.5 ± 55.22 vs. 927.42 ± 48.15, *p* < 0.001, ES = −2.02). The increasing trend of the maximum output power illustrated in the figure indicates that the FW group showed an increasing trend throughout the experimental intervention, whereas the PN group exhibited an increasing trend only in the first half of the experimental intervention (1–5 sessions) and a decreasing trend or a slight increment in the second half of the experiment. For both groups, linear sensing devices were employed to collect monitoring data; a linear sensor was used for the FW group, whereas a linear sensing device equipped with the pneumatic deep squat rack was used for the PN group. Although the working mechanism of both sensors is comparable in principle, it is unknown whether the specific device differences affect the output data.

### Pre–post comparisons

There were no significant differences between the FW and PN groups in the pre-test (Table 2).

The FW group underwent 6 weeks of traditional power barbell squatting. 30 m sprint time decreased by 0.12 s, but there was no significant difference in linear sprint speed before and after (*p* = 0.15, −2.4%, ES = 0.69) (Table 3). Participants showed significant improvements in jump performance, with CMJ-high (*p* < 0.01, 14%, ES = 1.82), CMJ-RP (*p* < 0.01, 8.7%, ES = 1.67), SJ-high (*p* = 0.04, 10.8%, ES = 1.02), SJ-RP (*p* = 0.02, 9.1%, ES = 1.20), DJ -high (*p* = 0.01, 14.68%, ES = 1.44), and DJ-RP (*p* < 0.01, 10.11%, ES = 1.59) (Table 3). However, the training effects of DJ-RSI (*p* = 0.65, 6.8%, ES = 0.21) and maximum strength (*p* = 0.14, 4.9%, ES = 0.72) were unclear (Table 3)

**TABLE 3:** Data analysis of the pre- and post-test indicators (FW).

Outcome	Baseline	Post-intervention	*p*-value	ES	SMD(90%CI)	Change score (% Δ)
**30-m sprint (s)**	**4.97 (0.19)**	**4.85 (0.19)**	**0.15**	**0.69**	**−0.58 (-1.05, -0.22)**	**−2.4%**
**CMJ-High (cm)**	**29.60 (3.29)**	**33.73 (3.54)**	**<0.01** ^**^	**1.82**	**1.16 (0.70, 1.82)**	**14%**
**CMJ-RP**	**40.92 (3.41)**	**44.49 (3.12)**	**<0.01** ^**^	**1.67**	**1.05 (0.66, 1.62)**	**8.70%**
**SJ-High (cm)**	**28.59 (4.38)**	**31.67 (3.54)**	**0.04** ^*^	**1.02**	**0.74 (0.30, 1.33)**	**10.80%**
**SJ-RP**	**39.77 (4.35)**	**43.4 (3.07)**	**0.02** ^*^	**1.2**	**0.92 (0 .42, 1.60)**	**9.10%**
**DJ-High (cm)**	**29.9 (4.47)**	**34.29 (3.08)**	**<0.01** ^**^	**1.44**	**1.09 (0.73, 1.66)**	**14.68%**
**DJ-RP**	**41.44 (4.43)**	**45.63 (2.65)**	**0.01** ^**^	**1.59**	**1.10 (0.72, 1.68)**	**10.11%**
**DJ-RSI**	**0.74 (0.25)**	**0.79 (0.17)**	**0.65**	**0.21**	**0.24 (-0.01, 0.55)**	**6.80%**
**maximal strength**	**82.00 (11.0)**	**86.00 (10.7)**	**0.14**	**0.72**	**0.35 (0.13, 0.64)**	**4.90%**

Table 3 Data are expressed as the mean ± standard deviation. ES = effect size (Hedgesʼ g); CI = confidence interval; SMD = standardized mean difference; Δ% = (post-baseline)/baseline × 100%. *Significant difference before and after training (*p* < 0.05); **Significant difference before and after training (*p* < 0.01). t-30 = 30-m short stroke time; CMJ-high = height of reverse vertical jump; CMJ-RP = relative power of reverse vertical jump; SJ-high = height of isometric squat jump; SJ-RP = relative power of isometric squat jump; DJ-high = height of falling jump; DJ-RP = relative power of falling jump; and DJ-RSI = reaction power index of falling jump.

The PN group underwent 6 weeks of power-based pneumatic resistance squat training. A 0.01-s reduction in 30 m sprint time was found (*p* = 0.99, 0.2%, ES < 0.01), and no significant differences were observed before and after training when linear velocity analysis was performed (Table 4). Participants’ jumping performance was higher in CMJ-high (*p* = 0.56, 4.2%, ES = 0.27), CMJ-RP (*p* = 0.56, 2.8%, ES = 0.27), SJ-high (*p* = 0.80, 3.4%, ES = 0.12), SJ-RP (*p* = 0.91, 1.8%, ES = 0.05), DJ-high (*p* = 0.96, 2%, ES = 0.02), DJ-RP (*p* = 1.00, 0.3%, ES < 0.01) and DJ-RSI (*p* = 0.94, 6%, ES = 0.04) (Table 4). However, a significant increase in maximal strength was noted after training (*p* < 0.01, 9.3%, ES = 1.67).

**TABLE 4:** Data analysis of the pre- and post-test indicators (PN).

Outcome	Baseline	Post-intervention	*p*-value	ES	SMD (90%CI)	Change score (%) Δ
30-m sprint (s)	**5.1** (**0.32**)	**5.09** (**0.32**)	**0.99**	**0.00**	**−0.04(-0.35,0.26)**	**0.2**
CMJ-High (cm)	**30.13** (**5.40**)	**31.4(5.90)**	**0.56**	**0.27**	**0.21(-0.01,0.48)**	**4.20**
CMJ-RP	**42.13** (**4.80**)	**43.3** (**5.14**)	**0.56**	**0.27**	**0.21(-0.06,0.53)**	**2.80**
SJ-High (cm)	**29.41** (**5.75**)	**30.4** (**5.12**)	**0.80**	**0.12**	**0.18(-0.06,0.45)**	**3.40**
SJ-RP	**41.55** (**4.91**)	**42.3** (**4.44**)	**0.91**	**0.05**	**0.16(-0.11,0.46)**	**1.80**
DJ-High (cm)	**30.66** (**6.39**)	**31.26** (**4.51**)	**0.96**	**0.02**	**0.1(-0.26,0.49)**	**2**
DJ-RP	**42.78** (**5.76**)	**42.89** (**4.39**)	**1**	**0.00**	**0.02(-0.29,0.33)**	**0.30**
DJ-RSI	**0.67** (**0.21**)	**0.71** (**0.21**)	**0.94**	**0.04**	**0.14(-0.06,0.11)**	**6**
maximal strength	**83.8** (**10.9**)	**91.6** (**9.42**)	**0.002** ^**^	**1.67**	**0.73** (**0.42**, **1.18**)	**9.30**

Data are expressed as the mean ± standard deviation. ES = effect size (Hedges ' g); CI = confidence interval; SMD = standardized mean difference; Δ% = (post-baseline)/baseline × 100%. *Significant difference before and after training (*p* < 0.05); **Significant difference before and after training (*p* < 0.01). t-30 = 30-m short stroke time; CMJ-high = height of reverse vertical jump; CMJ-RP = relative power of reverse vertical jump; SJ-high = height of isometric squat jump; SJ-RP = relative power of isometric squat jump; DJ-high = height of falling jump; DJ-RP = relative power of falling jump; and DJ-RSI = reaction power index of falling jump.

### Training effect

**FIGURE 3:**
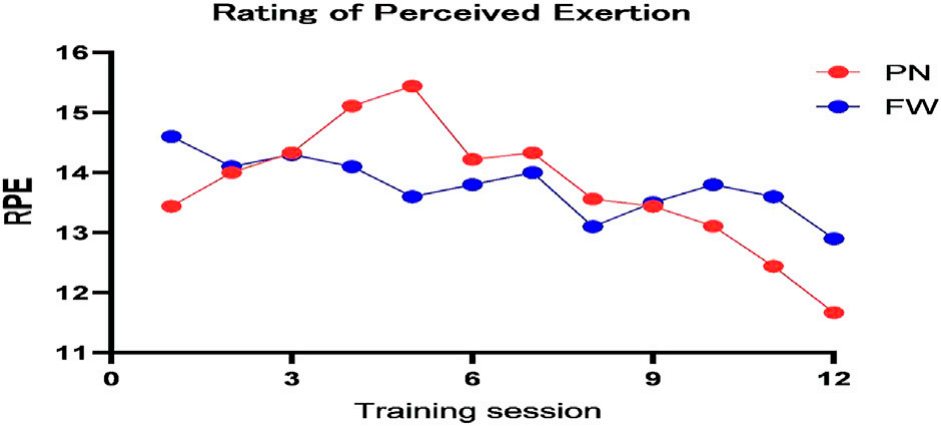
Rating of the perceived exertion change curve.

**FIGURE 4:**
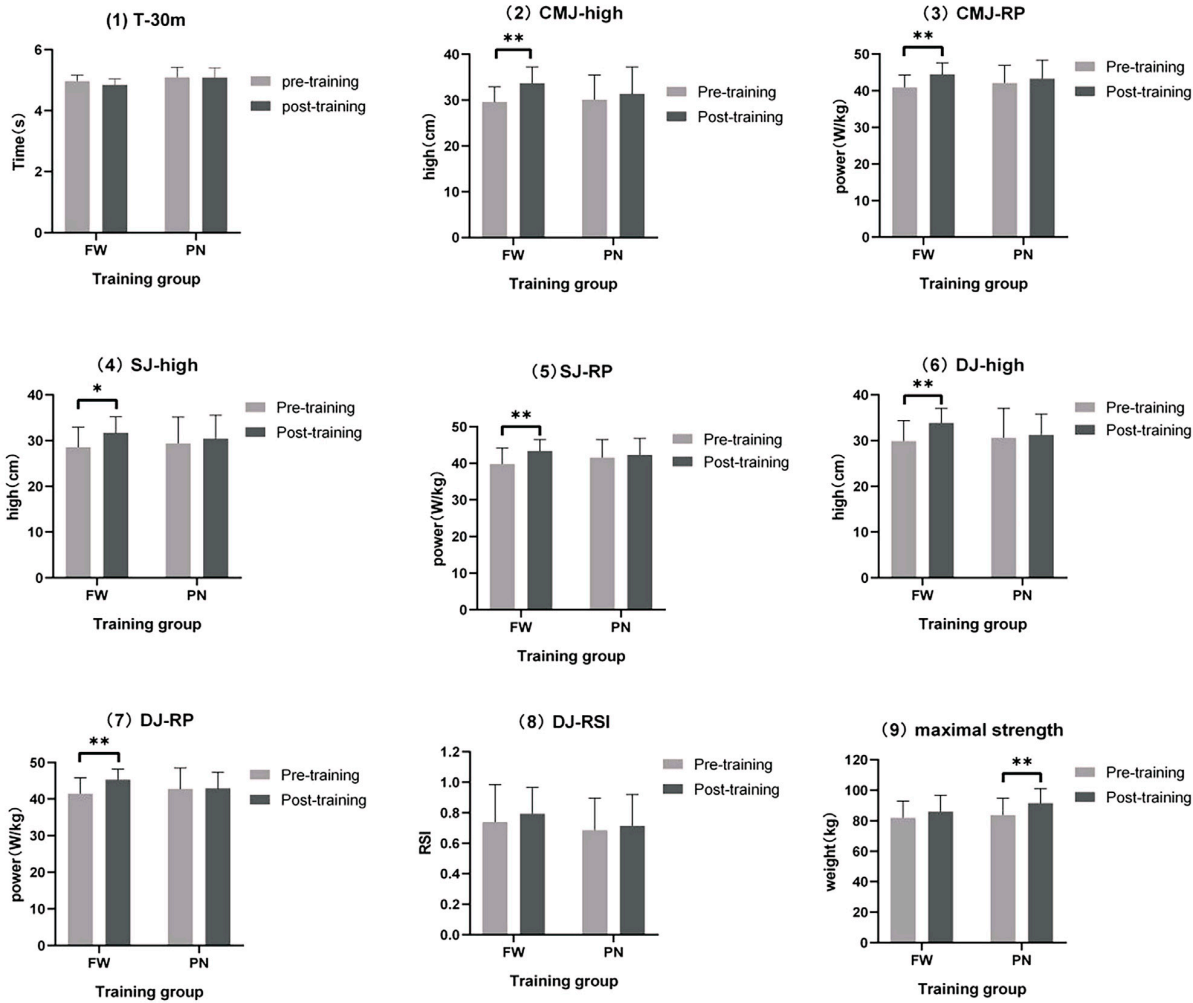
Mean changes in T-30M, CMJ-high, CMJ-RP, SJ-high, SJ-RP, DJ-high, DJ-RP, DJ-RSI, and maximal strength after 6 weeks of training. *Significant difference before and after (*p* < 0.05); **very significant difference before and after (*p* < 0.01).

No significant group differences were observed between the two groups in the tests of DJ-RSI (90.9%, likely trivial), SJ-high (74.6%, possibly trivial), SJ-RP (60.7%, possibly trivial), and maximal strength (70.8%, possibly trivial) (Table 5). Data analysis showed that strength training with free weight resistance had a positive effect on improving CMJ-high (58.7%, possibly FW), CMJ-RP (54%, possibly FW), DJ-high (58.7%, possibly FW), and DJ-RP (80.4%, likely FW) (Table 5). Furthermore, no significant group differences were observed in the gain of 30 m sprint time between the two groups (*p* = 0.16, ES = 0.11, possibly trivial). Although participants in the PN group showed an increase in jumping ability after the training intervention, the improvement was smaller than in the FW group (Figures 3, 4; Table 5). Thus, strength-based deep squat training with a free-weight resistance-based 70% 1 RM lift was more beneficial for athletes to improve explosive lower extremity strength in the vertical direction, a finding that is consistent with the study by David M. Frost et al.

**TABLE 5:** Data analysis of the pre- and post-test indicators (FW/PN).

Outcome	FW/PN mean difference	*p*-value	ES	SMD(90%CI)	Inference (MBI) B/T/H
30-m sprint (s)	**−0.1**	**0.16**	**0.11**	**−0.65** (**-1.54**, **0.11**)	**0.1/78.2/21.8**
					**possibly trivial**
CMJ-High (cm)	**2.88**	**0.03**	**0.26**	**1.07** (**0.31**, **2.04**)	**58.7/40.3/0**
					**possibly FW**
CMJ-RP	**2.45**	**0.04**	**0.24**	**1.01** (**0.25**, **1.97**)	**54/46/0**
					**possibly FW**
SJ-High (cm)	**2.08**	**0.15**	**0.12**	**0.66** (**-0.1**, **1.55**)	**25.2/74.6/0.2**
					**possibly trivial**
SJ-RP	**2.86**	**0.06**	**0.19**	**0.87** (**0.11**, **1.8**)	**39.3/60.7/0**
					**possibly trivial**
DJ-High (cm)	**3.79**	**0.02**	**0.27**	**1.10** (**0.34**, **2.07**)	**58.7/41.3/0**
					**possibly FW**
DJ-RP	**4.08**	**0.01**	**0.36**	**1.34** (**0.58**, **2.38**)	**80.4/19.6/0**
					**likely FW**
DJ-RSI	**0.03**	**0.67**	**0.01**	**0.19** (**-0.59**, **1.01**)	**7.6/90.9/1.5**
					**possibly trivial**
Maximal strength	**−3.78**	**0.12**	**0.14**	**−0.72** (**-1.62**, **0.04**)	**0.1/70.8/29.1**
					**possibly trivial**

Comparison of differences between the groups. Data are expressed as the mean ± standard deviations. ES = effect size (Cohen’s); CI = confidence interval; SMD = standardized mean difference; MBI = margin-based inference. FW/PN = group comparison of group differences between the free weight and pneumatic resistance groups. *Significant differences (*p* < 0.05) before and after training; T-30 = 30-m short stroke time; CMJ-high = height of reverse vertical jump; CMJ-RP = relative power of reverse vertical jump; SJ-high = height of isometric squat jump; SJ-RP = relative power of isometric squat jump; DJ-high = height of drop jump; DJ-RP = relative power of drop jump; DJ-RSI = reactive power index of the falling jump; and FW/PN = intergroup comparison between the free weight and the pneumatic resistance groups.

## Discussion

The primary objective of this study was to examine the effects of 6-week deep squat training with both resistance types on the performance of female youth judo athletes in linear velocity and vertical jump. In addition, the study indirectly assessed the differences in adaptation between the two resistance types to a 70% 1 RM load within 6 weeks of intervention training by monitoring the data.

### Adaptation differences between resistance types

Assessing the mean or peak of a variable may facilitate a more in-depth exploration of the total variation between conditions or resistance types, and assessing the shape of the curve may be useful for gaining insights into the magnitude of this variation (Cormie et al., 2008). As shown in Figure 2, the average maximum power output per training session for the PN group was greater than that of the FW group in the 6-week constant load model, which is consistent with the findings of Frost et al. (2008), Napoli et al. (2015), and Peltonen et al. (2013), indicating that except for 100% 1 RM conditions for training, the mean power output of the pneumatic device was significantly higher than the free weight resistance at any load intensity. Of note, although the weight of the load object is the main factor for generating resistance, the size of the load mass mainly causes the change in the state of motion because the load mass varies owing to changes in the position, velocity, and acceleration during the resistance process. Therefore, we attribute the following reasons for this discrepancy in output power:

1. When squatting with a conventional barbell, the muscles generate adequate force to overcome inertia and accelerate upward motion; however, the momentum of the barbell increases during the accelerated upward motion, which decreases the muscle work in the second part of the squatting process, eventually resulting in a decrease in power output (Lander et al., 1985).
2. The downward pull of the air pressure on the light barbell is the primary source of load during squatting with pneumatic resistance. This downward pull counteracts the effects of inertia and momentum in the squatting process, resulting in a consistently high level of muscle effort throughout the centripetal phase of the squat (i.e., the muscle effort required in the second half of the squatting process is not significantly reduced by the increases in inertia and momentum) (Frost et al., 2010). Thus, the decreasing magnitude of inertia and momentum caused the difference in power output between the two groups during the centripetal phase.

Figure 2 depicts the maximum power output variation curve for both groups of athletes during 12 sessions, with 6 training sessions serving as the midline, dividing the entire training intervention cycle. Both the PN and FW groups showed an upward trend in maximum output power early in the training cycle (first 6 sessions); the PN group leveled off in fluctuations later in the training cycle (second 6 sessions), which seemed to enter a bottleneck, whereas the FW group achieved the breakthrough late in the training cycle, displaying a plateau form.

Combined with the RPE scale used to evaluate athletes’ fatigue after training, the number of training sessions with stage peaks of maximum output power and fatigue index shown in Figures 2, 3 was used as an inflection point, indicating that the fatigue index of the PN group first showed an increasing trend and then a decreasing trend after training, while the fatigue index of the FW group gradually decreased in a fluctuating trend. The initial inflection point in the PN group occurred during the 5th training session and the same peak inflection point of the fatigue index was also observed in the fifth training session, whereas in the FW group, the peak inflection point occurred after that in the PN group. The maximum output power of both the PN and FW groups increased during the first five sessions; however, the fatigue index of the RPE showed significant differences, with the fatigue of the PN group increasing with the increase in the maximum output power and the fatigue of the FW group decreasing with training progression. Post-training fatigue decreased significantly in the PN group between the session 5 and session 10, with the decline being more significant than that in the FW group. The fatigue index dropped to its lowest value for both the pneumatic and free weight groups during sessions 10–12; however, the free weight group exceeded the baseline of maximum output power. This appears to reflect the way both resistance types adapt to neural fatigue, as both are the processes for adaptation to the corresponding load. The PN group entered the “power bottleneck” node significantly earlier than the FW group, and it is no coincidence that the RPE fatigue index also plummets after the node, thus indirectly reflecting the adaptation of pneumatic resistance precedes the free weight resistance.

It has been argued in previous studies that the neuromuscular system adapts differently depending on the level of fatigue (Gollhofer et al., 1987). As mentioned earlier, pneumatic resistance is a constant resistance, and athletes aim to adapt to the constant resistance to achieve high power output, which necessitates the recruitment of more neuromuscular and fast motor units, resulting in neurological fatigue. This may explain the increase in maximum power in the early part of the training session and the subsequent increase in fatigue in the PN group. Adaptation to free weight resistance is relatively slow, which is accompanied by a decrease in fatigue, and during the interval between training sessions, the body’s organs and systems function even beyond their original levels due to the exceeding compensation, thus providing an adequate material basis for the completion of the next training session (Wang and Hua, 2011) or even more than the original level, thus providing a sufficient material basis for completion of the next training. Under a fixed load, neural adaptation can recruit more neuromuscular and fast motor units, generating more force to resist the exogenous resistance; however, this is limited compared with the adaptation using free weights, which may explain the adaptation of the PN group before the FW group to break the “power bottleneck.” However, the aforementioned adaptation mechanisms for both resistance types are highly speculative and must be verified by further investigations at the biochemical level in participants.

### Linear velocity and vertical jump

As shown in Figure 4, after 6 weeks of intervention, there was no significant improvement in the linear velocity and vertical longitudinal jump indexes in the PN group; all indexes in the FW group showed significant improvement, except for DJ-RSI and T-30 M. Based on the experimental data, the free weight resistance type seems to result in more explosive power and optimal power output in young athletes at high loading weights (70% 1 RM) than the pneumatic resistance type during short-term training interventions; however, the improvement in reaction power was not significant. The results are broadly analogous to those of David M. Frost et al., (Peltonen et al., 2013) who trained 18 adults with at least 3 years of resistance expertise during an 8-week intervention, with 18 participants allocated each to the pneumatic resistance training and free weight training groups. The power output of both the PN and FW groups was significantly improved after 8 weeks of training intervention (FW: *p* = 0.046, ES = 0.62: PN: *p* = 0.04, ES = 1.08). Additionally, analysis of the training monitoring indices during the complete training intervention indicated a significant change in the power output of the PN group at light loads (15%–45%1 RM), while the change in the FW group was observed at heavy loads (60%–90%1 RM). Furthermore, Heikki Peltonen et al., (Frost et al., 2008). Reported that the difference between the two machines, pneumatic resistance, and conventional barbell, decreased with an increase in the load because increasing the weight reduced the effect of momentum and inertia on free weight training.

We summarize the reasons for differences between the two resistance types in the short-term intervention as follows. First, the resistance with the barbell is highest at the beginning of the centripetal phase of the squat but progressively falls owing to the presence of inertial acceleration, whereas the resistance is relatively stable throughout the squat due to the pneumatic resistance characteristics. According to the law of conservation of momentum (*p* = m × v) and the force-velocity curve, an increase in load weight reduces the momentum exerted on the barbell during the participant’s squat, thereby reducing the difference in resistance between the two resistance types during the squat. Furthermore, according to the analysis of the vertical jump resistance pattern, the body overcomes its weight upward during the centripetal phase, and once it starts moving, the body gains inertia and momentum due to acceleration, reducing the resistance to upward movement, consistent with the resistance pattern of the barbell squat. Consequently, one of the reasons for the difference between the two resistance types in the vertical jump test may be the consistency of the resistance pattern; additionally, results of the experimental data analysis indicated that the improvement in sprint time did not differ significantly between both groups, which may be due to the difference in movement patterns because the vertical jump is similar to the movement pattern of the deep squat and no sprinting exercises were performed during the experiment.

**FIGURE 5:**
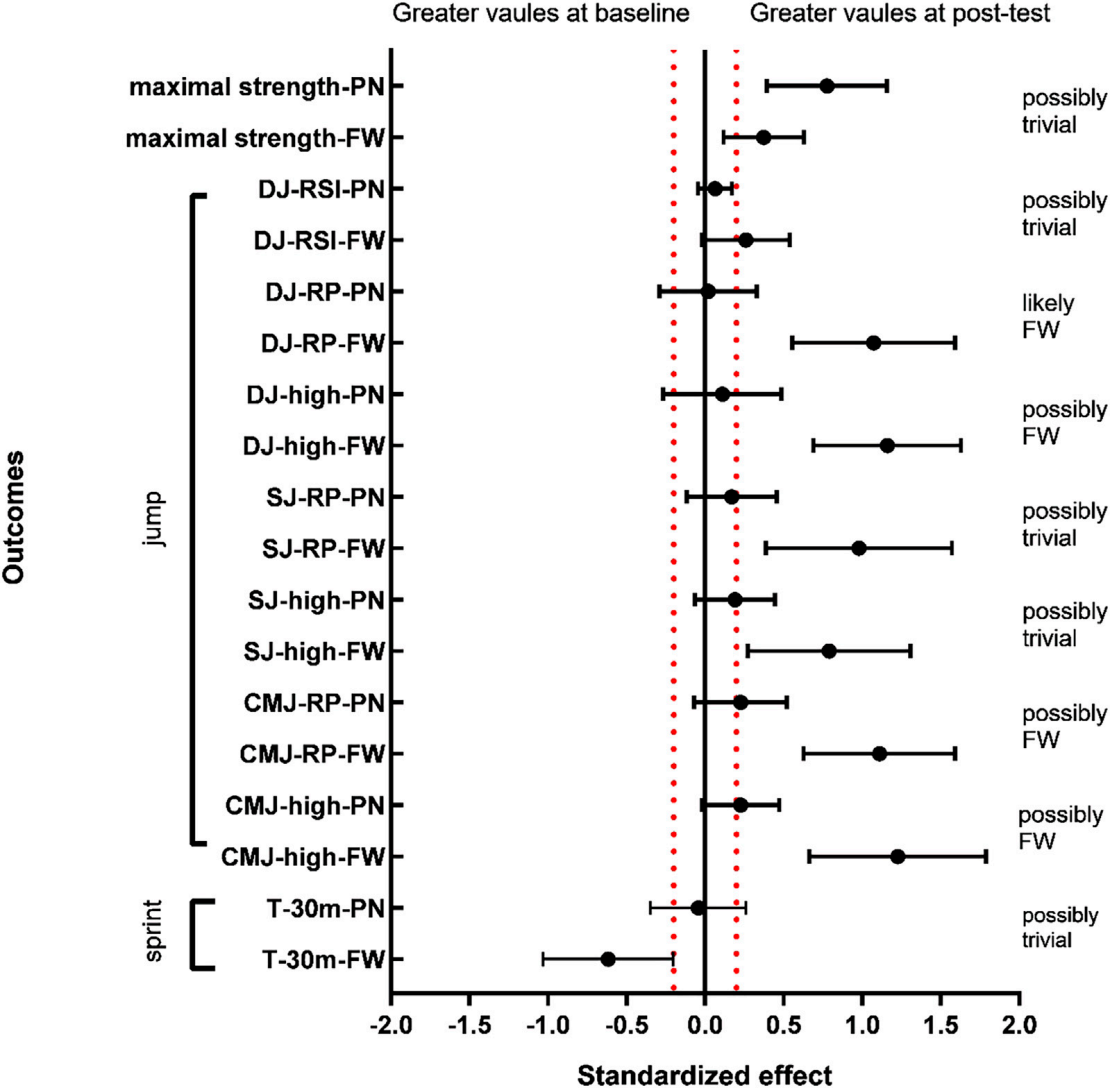
Normalized results (90% confidence interval) for all outcomes at the baseline and post-training for the FW and PN groups. T-30 = 30-m short stroke time; CMJ-high = reverse vertical jump height; CMJ-RP = relative power of reverse vertical jump; SJ-high = isometric squat jump height; SJ-RP = relative power of isometric squat jump; DJ-high = drop jump height; DJ-RP = relative power of drop jump; and DJ-RSI = reaction power index of drop jump.

Second, as shown in Figure 5, the FW group may outperform the aerodynamic resistance group in the CMJ and DJ tests. As per the force-velocity curve, the greater the weight of the load, the greater is the force required to overcome the resistance and the slower is the speed. Frost DM et al., (Frost et al., 2010) reported that a high angular velocity facilitates the generation of high average power during explosive motion, and Heikki Peltonen et al., (Frost et al., 2008). Demonstrated a significant increase in angular velocity from 60% to 140% of the knee angle for all loading intensities during free weight squats; however, no significant changes in angular velocity of the knee joint were observed during pneumatic resistance training. At 70% 1 RM load weight, pneumatic resistance cannot play the action speed of a low load weight and complete the squat at a relatively low speed, which contradicts pneumatic resistance’s unique advantage of constant and high speed. Hence, the difference in angular velocity of the two resistance mode training interventions to complete the squat at a loading weight of 70% 1 RM is responsible for the difference in performance in the CMJ and DJ tests, indicating that free weight resistance is superior to pneumatic resistance in increasing the athlete’s slow stretch compounding capacity and fast stretch compounding capacity.

Interestingly, during each intervention, individuals were verbally encouraged to ensure the maximum voluntary contraction for each squat. According to the law of conservation of momentum (*p* = m × v), the mass of the barbell is constant; the momentum gained by the barbell increases with an increase in the speed, and the resistance induced after the start decreases with an increase in momentum. Short-term free weight resistance training possibly allows an individual to adapt to the characteristics of the resistance plunge, and the magnitude of the resistance plunge increases as the start acceleration increases, and this adaptation may make an individual more inclined toward their neural impulses on the start acceleration phase; the pneumatic device’s constant resistance characteristics necessitate maintaining a constant force at each joint angle to ensure the output power of each squat. Thus, participants’ neural adaptation to the characteristics of their resistance type may account for the difference in initial strength capacity, which explains the slightly better performance of the conventional barbell group than the pneumatic resistance group in the SJ test in the present study, although this difference is very slight on the MBI (Figure 5).

Furthermore, no significant difference in DJ-RSI was observed before and after this experiment (Figure 4). At 70% 1 RM counterweight, the type of resistance appeared to have a substantial effect on the reactive strength index. The explanation might be that no Plyometric or neural response exercises (e.g., deep jump, reaction jump, etc.) were scheduled during the training cycle.

### Maximum strength

This might also explain better performance of the pneumatic resistance group performed slightly better than the free weight resistance group in the maximum strength test since the PN group increased the power output of each squat (Figure 5). This result is consistent with the experimental results of David M. Frost (Frost et al., 2016), as discovered through a review of their experiments: they conducted an 8-week comparative intervention experiment by using transformed loads [one week including strength (80%–95% 1 RM) and power (30%–45% 1 RM)], and the maximum strength gain in the PN group after the intervention was not significantly different from that in the FW group. However, as shown in Figure 5, this experiment identified the pneumatic resistance group as the potentially beneficial group (possibly trivial, 29.1%), although this difference was small; however, in the study by David M. Frost et al. this group was not reported as potentially beneficial (probably trivial, > 37%). For a 6-week comparison experiment, a fixed 70% 1 RM weight was used, and the large load weight could decrease the difference in resistance between the two resistance types during the squatting process, thus decreasing the difference in the power output of the body per squat, which may account for the non-significant difference in the maximum power gain between the two groups after training. Even though the resistance difference between the two groups is reduced with an increase in the weights, the resistance characteristics of the pneumatic resistance can still provide greater output power per squat than the free weight resistance, which may explain why the pneumatic resistance group is potentially beneficial. The maximum strength gain from pneumatic resistance appears to be slightly superior to that for free weight resistance for young athletes at 70% 1 RM load weight. Notably, no significant gain was noted in the pneumatic resistance group in the vertical jump test, indicating that the maximum strength gain from pneumatic resistance does not seem to translate better to athletic performance.

### Practical applications

Different resistance types have potential strengths and limitations, and no training stimulus can be employed singly for all training purposes. Within each resistance type, different weights, movement speeds, intervals, and movement choices all play a role in their unique training stimulus. Although the PN group had a higher maximum output than the FW group throughout the training intervention, the FW group outperformed the PN group in the vertical jump test, and the deep squat training alone appeared to have a negligible improvement in linear velocity. Therefore, the efficacy of training in smoothly improving the athletic performance of athletes is a factor that should be considered by coaches or researchers. In addition, the adaptation of the body to pneumatic resistance seems to be faster than that to free weight resistance, and coaches may use this knowledge to precise identify the athletes’ bottleneck in the training process and modify the load intensity in time to generate new adaptations. Short-term squatting exercises with pneumatic resistance and larger load weights appear to be more conducive to maximum leg strength gains. To maximize the athletes’ physical strength gain, coaches should utilize the resistance properties of various resistance types to adjust the training program for different training phases of different sports

## Limitations

The rest period for adolescent athletes is limited, and since an increase in training intensity after the rest period may have an effect on the results of the experiment, it is not possible to determine if a longer experimental period would have an effect on the results of this experiment. Follow-up studies with longer experimental periods and involving mid-experimental tests are needed to observe the temporal effects of the training intervention. In addition, the small overall sample size is one of the limitations of this study because the subjects were high-level adolescent female judo athletes and there was uncontrollable attrition in the screening and intervention process. Therefore, further studies involving participants of different age groups, gender, weight, and years of training are needed.

## Data Availability

The original contributions presented in the study are included in the article/Supplementary Material, further inquiries can be directed to the corresponding author.
